# Associations between parental history of Alzheimer's disease and plasma markers of immunity in a multi‐ethnic community cohort of middle‐aged adults

**DOI:** 10.1002/alz70856_103420

**Published:** 2025-12-26

**Authors:** Edric D. Winford, Benjamin D. Huber, Jessica Mazen, Adam Brickman, Jennifer J. Manly

**Affiliations:** ^1^ Columbia University, New York, NY, USA; ^2^ Taub Institute for Research on Alzheimer's Disease and the Aging Brain, Columbia University, New York, NY, USA; ^3^ Columbia University Irving Medical Center, New York, NY, USA; ^4^ Department of Neurology, Vagelos College of Physicians and Surgeons, Columbia University, New York, NY, USA; ^5^ Department of Neurology, Columbia University, New York, NY, USA

## Abstract

**Background:**

Peripheral inflammation is a known contributor to Alzheimer's disease (AD) risk and progression. As aging is associated with increasing inflammation that may precede AD onset, individuals with a parental history of AD, a risk factor that increases lifetime AD risk, may have inflammatory profiles that reflect their risk. This study examines whether having a parent with AD is associated with increased peripheral inflammation in middle‐aged adults. We hypothesize that individuals with parental history of AD will have higher levels of pro‐inflammatory cytokines compared with those without a parental history of AD across racial and ethnic groups.

**Method:**

Participants comprised 1,043 non‐Hispanic White, non‐Hispanic Black, and Hispanic men and women from the Offspring Study who are the adult children of participants in the Washington Heights Inwood Columbia Aging Project. Parental AD status was determined by a diagnostic consensus conference. Plasma chemokine and cytokine concentrations were assayed with Luminex technology. were used for the associations between parental AD status and inflammatory cytokines, adjusting for age and sex, and interaction models were used to determine if associations differed by age, sex at birth, race and ethnicity, or APOE genotype.

**Result:**

Participants with a parental history of AD (*n* = 660, mean age=54, 65% women) had higher levels of Eotaxin (log‐diff: 0.09, 95% CI: 0.01,.0.15) and lower levels of G‐CSF (‐0.2, 95% CI: ‐0.35,‐0.04), VEGF‐A (‐0.16, 95% CI: ‐0.3,‐0.01), and IL‐27 (‐0.07, 95% CI: ‐0.15,0.0) compared with those without a parental history of AD (*n* = 383, mean age=60, 71% women). IL‐18 levels were significantly higher in Black individuals with a parental history of AD compared to Black individuals without, while White individuals with a parental history of AD had higher levels of EGF compared to White individuals without. Women with a parent history of AD had higher levels of IFna2, IL‐12p70, sCD40L, and IL‐18 compared to women without parental AD history.

**Conclusion:**

Parental history of AD is associated with elevated markers of peripheral inflammation. These associations vary across sex and race and ethnicity.

